# Supplementary material for: Optimizing Motor Imagery Parameters for Robotic Arm Control by Brain-Computer Interface
Source: Brain Sci. 2022 Jun 26;12(7):833. doi: 10.3390/brainsci12070833 (PMC9313178; doi:10.3390/brainsci12070833)
Supplement: Supplementary file 1 [file brainsci-12-00833-s001.zip › brainsci-1721667-supplementary.pdf]

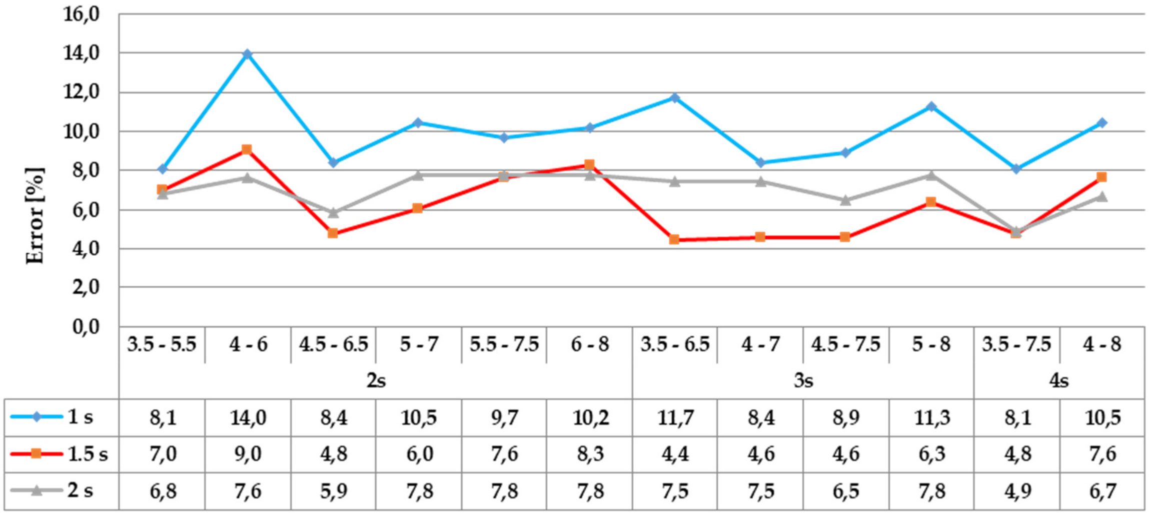

	3.5 - 5.5	4 - 6	4.5 - 6.5	5 - 7	5.5 - 7.5	6 - 8	3.5 - 6.5	4 - 7	4.5 - 7.5	5 - 8	3.5 - 7.5	4 - 8
	2s						3s				4s	
1 s	8,1	14,0	8,4	10,5	9,7	10,2	11,7	8,4	8,9	11,3	8,1	10,5
1.5 s	7,0	9,0	4,8	6,0	7,6	8,3	4,4	4,6	4,6	6,3	4,8	7,6
2 s	6,8	7,6	5,9	7,8	7,8	7,8	7,5	7,5	6,5	7,8	4,9	6,7

Figure S1. Subject S1, Mean errors for different CSP time windows and three variance time windows

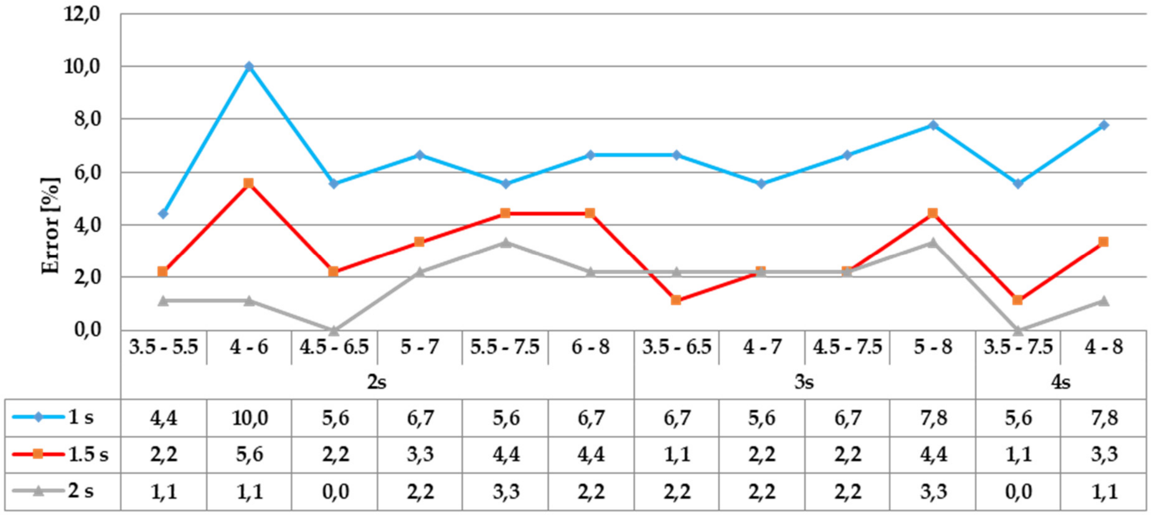

	3.5 - 5.5	4 - 6	4.5 - 6.5	5 - 7	5.5 - 7.5	6 - 8	3.5 - 6.5	4 - 7	4.5 - 7.5	5 - 8	3.5 - 7.5	4 - 8
	2s						3s				4s	
1 s	4,4	10,0	5,6	6,7	5,6	6,7	6,7	5,6	6,7	7,8	5,6	7,8
1.5 s	2,2	5,6	2,2	3,3	4,4	4,4	1,1	2,2	2,2	4,4	1,1	3,3
2 s	1,1	1,1	0,0	2,2	3,3	2,2	2,2	2,2	2,2	3,3	0,0	1,1

Figure S2. Subject S1, Minimal errors for different CSP time windows and three variance time windows

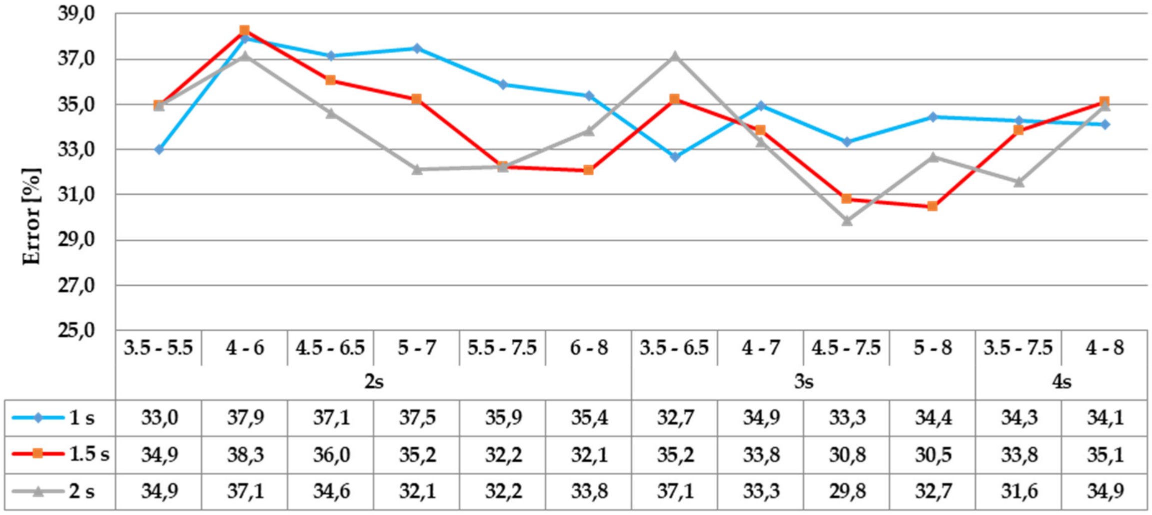

	3.5 - 5.5	4 - 6	4.5 - 6.5	5 - 7	5.5 - 7.5	6 - 8	3.5 - 6.5	4 - 7	4.5 - 7.5	5 - 8	3.5 - 7.5	4 - 8
	2s						3s				4s	
1 s	33,0	37,9	37,1	37,5	35,9	35,4	32,7	34,9	33,3	34,4	34,3	34,1
1.5 s	34,9	38,3	36,0	35,2	32,2	32,1	35,2	33,8	30,8	30,5	33,8	35,1
2 s	34,9	37,1	34,6	32,1	32,2	33,8	37,1	33,3	29,8	32,7	31,6	34,9

Figure S3. Subject S2, Mean errors for different CSP time windows and three variance time windows

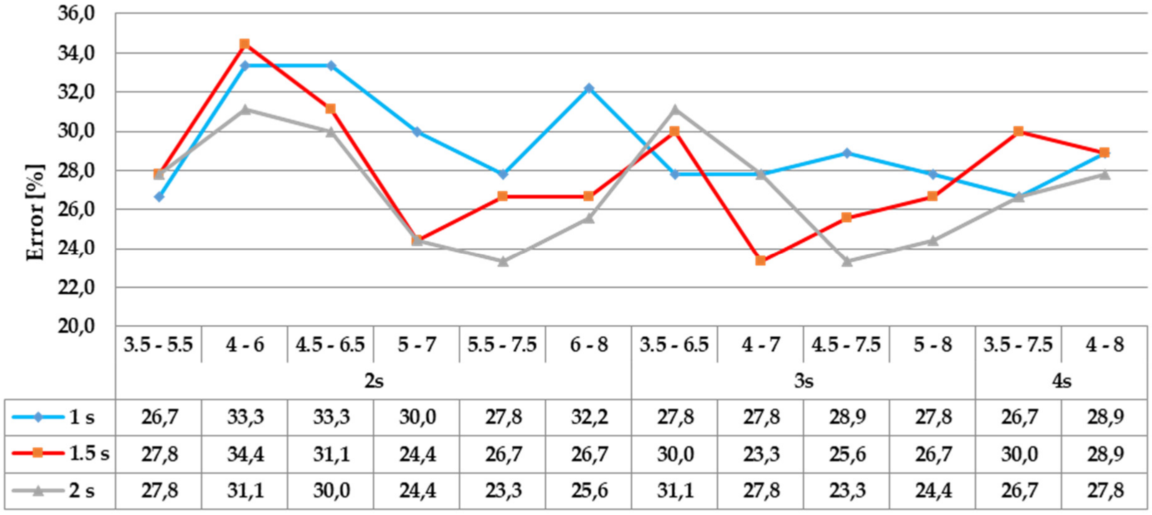

	3.5 - 5.5	4 - 6	4.5 - 6.5	5 - 7	5.5 - 7.5	6 - 8	3.5 - 6.5	4 - 7	4.5 - 7.5	5 - 8	3.5 - 7.5	4 - 8
	2s						3s				4s	
1 s	26,7	33,3	33,3	30,0	27,8	32,2	27,8	27,8	28,9	27,8	26,7	28,9
1.5 s	27,8	34,4	31,1	24,4	26,7	26,7	30,0	23,3	25,6	26,7	30,0	28,9
2 s	27,8	31,1	30,0	24,4	23,3	25,6	31,1	27,8	23,3	24,4	26,7	27,8

Figure S4. Subject S2, Minimal errors for different CSP time windows and three variance time windows

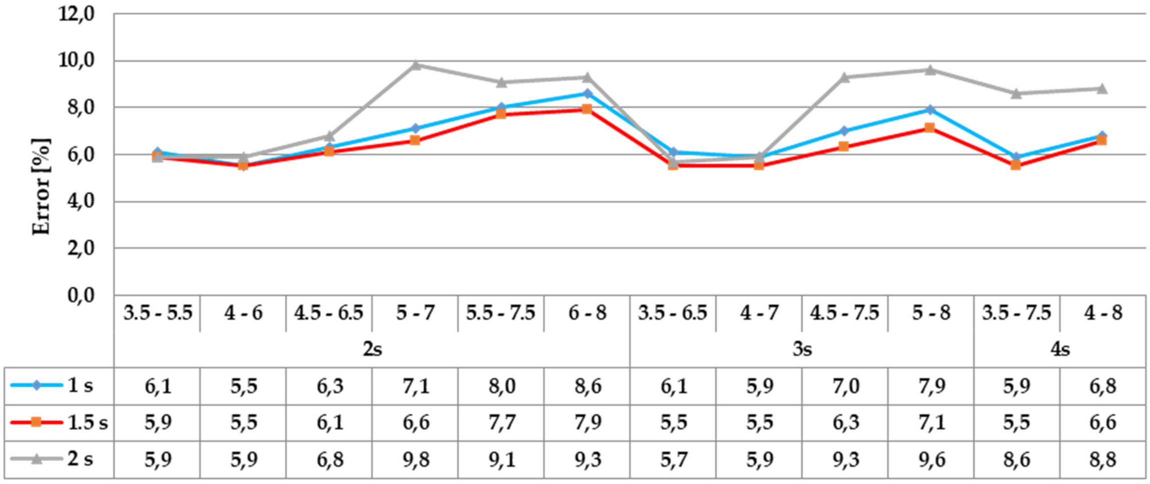

	3.5 - 5.5	4 - 6	4.5 - 6.5	5 - 7	5.5 - 7.5	6 - 8	3.5 - 6.5	4 - 7	4.5 - 7.5	5 - 8	3.5 - 7.5	4 - 8
	2s						3s				4s	
1 s	6,1	5,5	6,3	7,1	8,0	8,6	6,1	5,9	7,0	7,9	5,9	6,8
1.5 s	5,9	5,5	6,1	6,6	7,7	7,9	5,5	5,5	6,3	7,1	5,5	6,6
2 s	5,9	5,9	6,8	9,8	9,1	9,3	5,7	5,9	9,3	9,6	8,6	8,8

Figure S5. Subject S3, Mean errors for different CSP time windows and three variance time windows

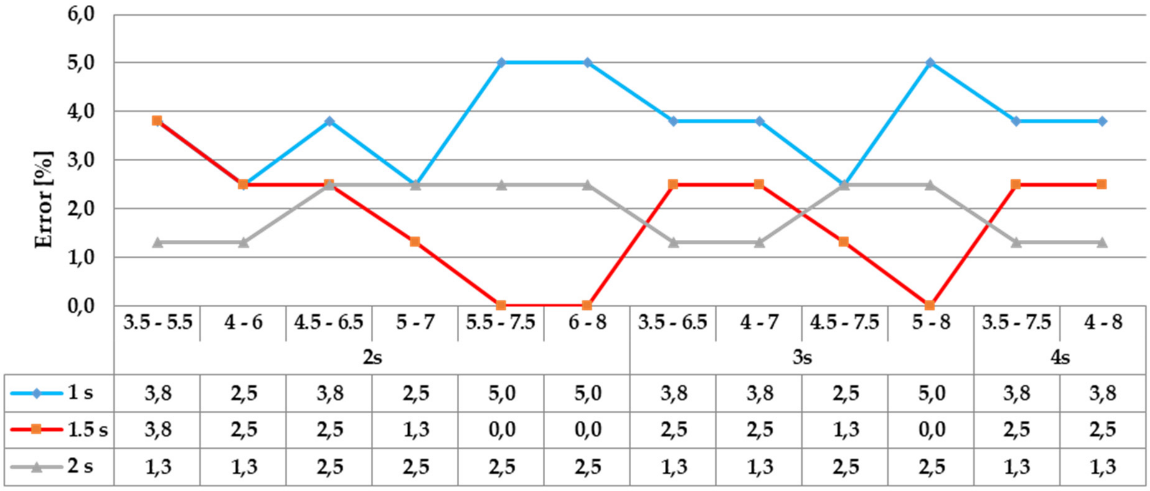

	3.5 - 5.5	4 - 6	4.5 - 6.5	5 - 7	5.5 - 7.5	6 - 8	3.5 - 6.5	4 - 7	4.5 - 7.5	5 - 8	3.5 - 7.5	4 - 8
	2s						3s				4s	
1 s	3,8	2,5	3,8	2,5	5,0	5,0	3,8	3,8	2,5	5,0	3,8	3,8
1.5 s	3,8	2,5	2,5	1,3	0,0	0,0	2,5	2,5	1,3	0,0	2,5	2,5
2 s	1,3	1,3	2,5	2,5	2,5	2,5	1,3	1,3	2,5	2,5	1,3	1,3

Figure S6. Subject S3, Minimal errors for different CSP time windows and three variance time windows

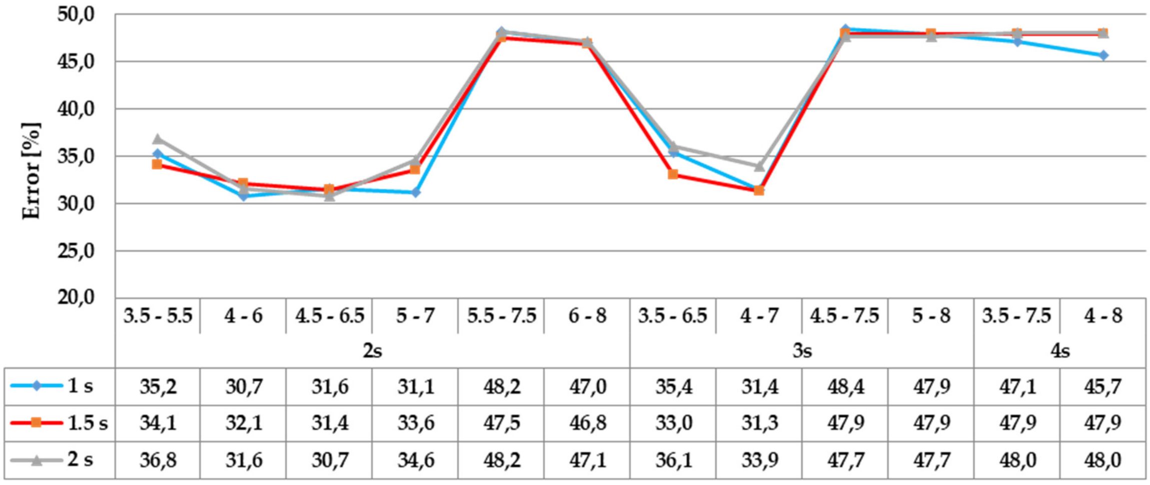

	3.5 - 5.5	4 - 6	4.5 - 6.5	5 - 7	5.5 - 7.5	6 - 8	3.5 - 6.5	4 - 7	4.5 - 7.5	5 - 8	3.5 - 7.5	4 - 8
	2s						3s				4s	
1 s	35,2	30,7	31,6	31,1	48,2	47,0	35,4	31,4	48,4	47,9	47,1	45,7
1.5 s	34,1	32,1	31,4	33,6	47,5	46,8	33,0	31,3	47,9	47,9	47,9	47,9
2 s	36,8	31,6	30,7	34,6	48,2	47,1	36,1	33,9	47,7	47,7	48,0	48,0

Figure S7. Subject S4, Mean errors for different CSP time windows and three variance time windows

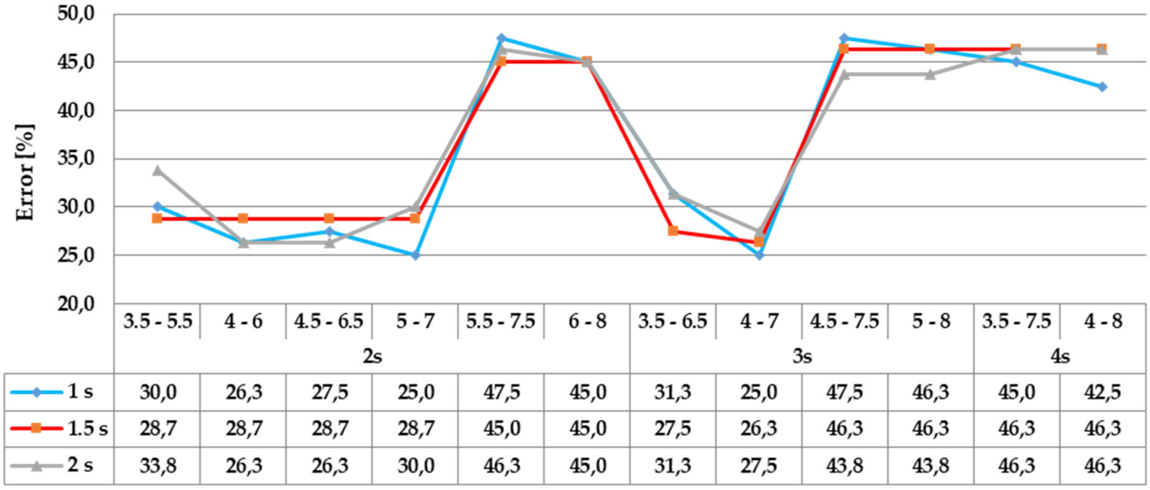

	3.5 - 5.5	4 - 6	4.5 - 6.5	5 - 7	5.5 - 7.5	6 - 8	3.5 - 6.5	4 - 7	4.5 - 7.5	5 - 8	3.5 - 7.5	4 - 8
	2s						3s				4s	
1 s	30,0	26,3	27,5	25,0	47,5	45,0	31,3	25,0	47,5	46,3	45,0	42,5
1.5 s	28,7	28,7	28,7	28,7	45,0	45,0	27,5	26,3	46,3	46,3	46,3	46,3
2 s	33,8	26,3	26,3	30,0	46,3	45,0	31,3	27,5	43,8	43,8	46,3	46,3

Figure S8. Subject S4, Minimal errors for different CSP time windows and three variance time windows

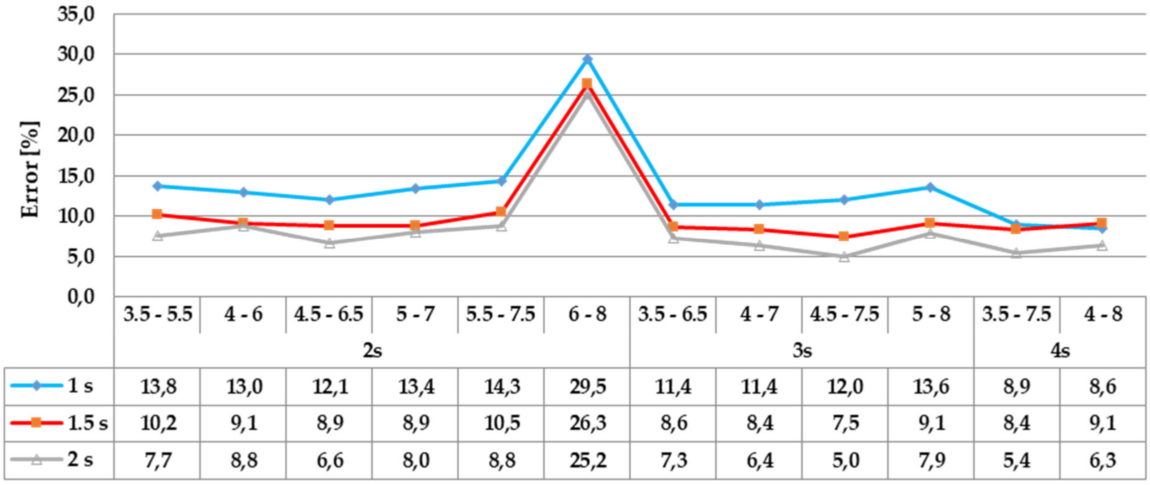

	3.5 - 5.5	4 - 6	4.5 - 6.5	5 - 7	5.5 - 7.5	6 - 8	3.5 - 6.5	4 - 7	4.5 - 7.5	5 - 8	3.5 - 7.5	4 - 8
	2s						3s				4s	
1 s	13,8	13,0	12,1	13,4	14,3	29,5	11,4	11,4	12,0	13,6	8,9	8,6
1.5 s	10,2	9,1	8,9	8,9	10,5	26,3	8,6	8,4	7,5	9,1	8,4	9,1
2 s	7,7	8,8	6,6	8,0	8,8	25,2	7,3	6,4	5,0	7,9	5,4	6,3

Figure S9. Subject S5, Mean errors for different CSP time windows and three variance time windows

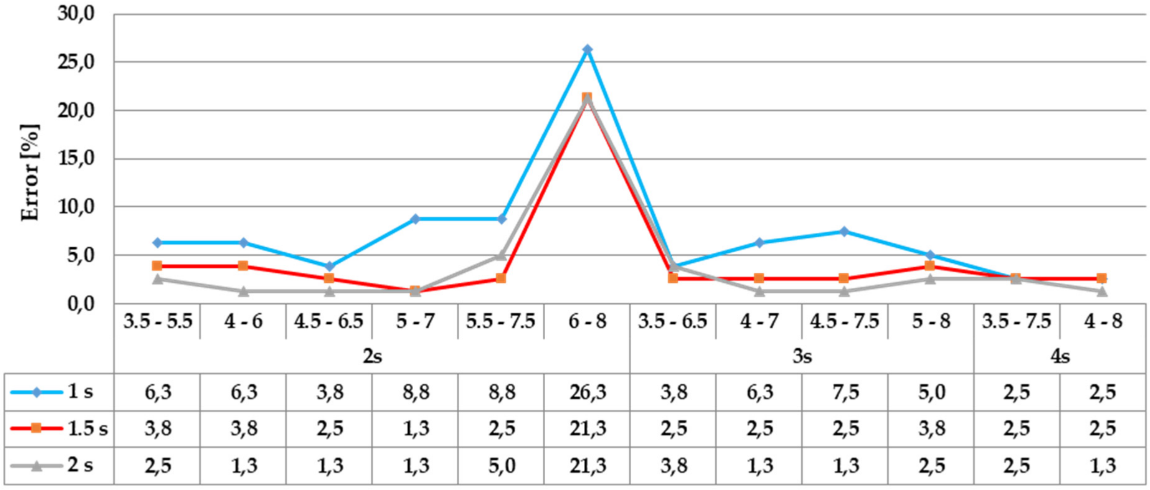

	3.5 - 5.5	4 - 6	4.5 - 6.5	5 - 7	5.5 - 7.5	6 - 8	3.5 - 6.5	4 - 7	4.5 - 7.5	5 - 8	3.5 - 7.5	4 - 8
	2s						3s				4s	
1 s	6,3	6,3	3,8	8,8	8,8	26,3	3,8	6,3	7,5	5,0	2,5	2,5
1.5 s	3,8	3,8	2,5	1,3	2,5	21,3	2,5	2,5	2,5	3,8	2,5	2,5
2 s	2,5	1,3	1,3	1,3	5,0	21,3	3,8	1,3	1,3	2,5	2,5	1,3

Figure S10. Subject S5, Minimal errors for different CSP time windows and three variance time windows

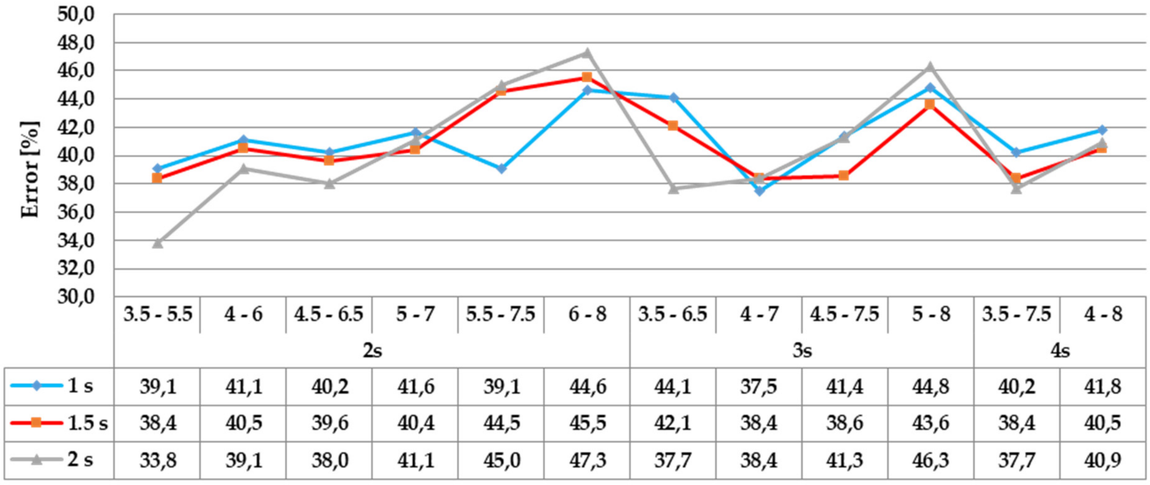

	3.5 - 5.5	4 - 6	4.5 - 6.5	5 - 7	5.5 - 7.5	6 - 8	3.5 - 6.5	4 - 7	4.5 - 7.5	5 - 8	3.5 - 7.5	4 - 8
	2s						3s				4s	
1 s	39,1	41,1	40,2	41,6	39,1	44,6	44,1	37,5	41,4	44,8	40,2	41,8
1.5 s	38,4	40,5	39,6	40,4	44,5	45,5	42,1	38,4	38,6	43,6	38,4	40,5
2 s	33,8	39,1	38,0	41,1	45,0	47,3	37,7	38,4	41,3	46,3	37,7	40,9

Figure S11. Subject S6, Mean errors for different CSP time windows and three variance time windows

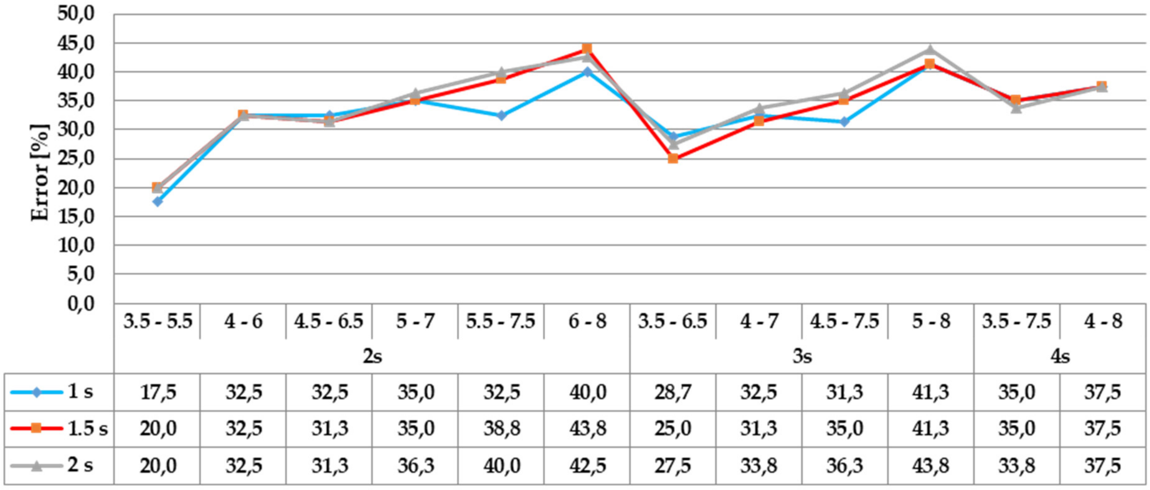

	3.5 - 5.5	4 - 6	4.5 - 6.5	5 - 7	5.5 - 7.5	6 - 8	3.5 - 6.5	4 - 7	4.5 - 7.5	5 - 8	3.5 - 7.5	4 - 8
	2s						3s				4s	
1 s	17,5	32,5	32,5	35,0	32,5	40,0	28,7	32,5	31,3	41,3	35,0	37,5
1.5 s	20,0	32,5	31,3	35,0	38,8	43,8	25,0	31,3	35,0	41,3	35,0	37,5
2 s	20,0	32,5	31,3	36,3	40,0	42,5	27,5	33,8	36,3	43,8	33,8	37,5

Figure S12. Subject S6, Minimal errors for different CSP time windows and three variance time windows

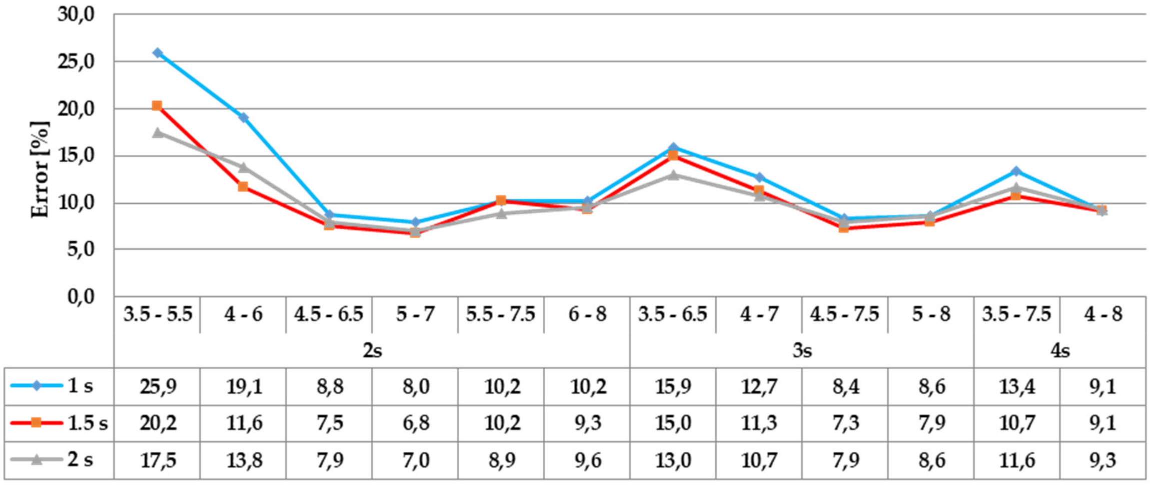

	3.5 - 5.5	4 - 6	4.5 - 6.5	5 - 7	5.5 - 7.5	6 - 8	3.5 - 6.5	4 - 7	4.5 - 7.5	5 - 8	3.5 - 7.5	4 - 8
	2s						3s				4s	
1 s	25,9	19,1	8,8	8,0	10,2	10,2	15,9	12,7	8,4	8,6	13,4	9,1
1.5 s	20,2	11,6	7,5	6,8	10,2	9,3	15,0	11,3	7,3	7,9	10,7	9,1
2 s	17,5	13,8	7,9	7,0	8,9	9,6	13,0	10,7	7,9	8,6	11,6	9,3

Figure S13. Subject S7, Mean errors for different CSP time windows and three variance time windows

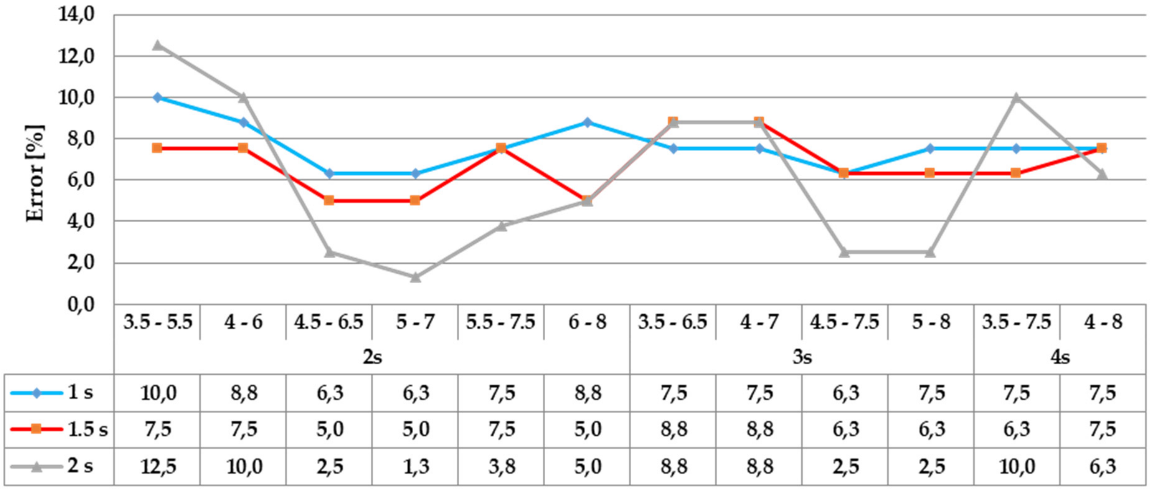

	3.5 - 5.5	4 - 6	4.5 - 6.5	5 - 7	5.5 - 7.5	6 - 8	3.5 - 6.5	4 - 7	4.5 - 7.5	5 - 8	3.5 - 7.5	4 - 8
	2s						3s				4s	
1 s	10,0	8,8	6,3	6,3	7,5	8,8	7,5	7,5	6,3	7,5	7,5	7,5
1.5 s	7,5	7,5	5,0	5,0	7,5	5,0	8,8	8,8	6,3	6,3	6,3	7,5
2 s	12,5	10,0	2,5	1,3	3,8	5,0	8,8	8,8	2,5	2,5	10,0	6,3

Figure S14. Subject S7, Minimal errors for different CSP time windows and three variance time windows
